# A Singular Spectrum Analysis-Based Data-Driven Technique for the Removal of Cardiogenic Oscillations in Esophageal Pressure Signals

**DOI:** 10.1109/JTEHM.2020.3012926

**Published:** 2020-07-30

**Authors:** Sourav Kumar Mukhopadhyay, Michael Zara, Irene Telias, Lu Chen, Rémi Coudroy, Takeshi Yoshida, Laurent Brochard, Sridhar Krishnan

**Affiliations:** 1 Department of Electrical, Computer, and Biomedical EngineeringRyerson University 7984 TorontoONM5B 2K3Canada; 2 Institute for Biomedical Engineering, Science, and Technology (iBEST), Ryerson University 7984 TorontoONM5B 2K3Canada; 3 Keenan Research Centre, Li Ka Shing Knowledge Institute, St. Michael’s HospitalTorontoONM5B 1T8Canada; 4 Intensive Care UnitOsaka University Hospital 157453 Osaka565-0871Japan

**Keywords:** Cardiogenic oscillation, data-driven technique, esophageal pressure signal, mean opinion score, mechanical ventilation, singular spectrum analysis

## Abstract

*Objective:* Assessing the respiratory and lung mechanics of the patients in intensive care units is of utmost need in order to guide the management of ventilation support. The esophageal pressure ($\boldsymbol {P}_{ \boldsymbol {eso}}$) signal is a minimally invasive measure, which portrays the mechanics of the lung and the pattern of breathing. Because of the close proximity of the lung to the beating heart inside the thoracic cavity, the $\boldsymbol {P}_{ \boldsymbol {eso}}$ signals always get contaminated with that of the oscillatory-pressure-signal of the heart, which is known as the cardiogenic oscillation ($\boldsymbol {CGO}$) signal. However, the area of research addressing the removal of $\boldsymbol {CGO}$ from $\boldsymbol {P}_{ \boldsymbol {eso}}$ signal is still lagging behind. *Methods and results:* This paper presents a singular spectrum analysis-based high-efficient, adaptive and robust technique for the removal of $\boldsymbol {CGO}$ from $\boldsymbol {P}_{ \boldsymbol {eso}}$ signal utilizing the inherent periodicity and morphological property of the $\boldsymbol {P}_{ \boldsymbol {eso}}$ signal. The performance of the proposed technique is tested on $\boldsymbol {P}_{ \boldsymbol {eso}}$ signals collected from the patients admitted to the intensive care unit, cadavers, and also on synthetic $\boldsymbol {P}_{ \boldsymbol {eso}}$ signals. The efficiency of the proposed technique in removing $\boldsymbol {CGO}$ from the $\boldsymbol {P}_{ \boldsymbol {eso}}$ signal is quantified through both qualitative and quantitative measures, and the mean opinion scores of the denoised $\boldsymbol {P}_{ \boldsymbol {eso}}$ signal fall under the categories ‘very good’ as per the subjective measure. *Conclusion and clinical impact:* The proposed technique: (1) does not follow any predefined mathematical model and hence, it is data-driven, (2) is adaptive to the sampling rate, and (3) can be adapted for denoising other biomedical signals which exhibit periodic or quasi-periodic nature.

## Graphical abstract

The aim and motivation behind the proposed research work is to design a high-performance data-driven cardiogenic-oscillation removal technique from esophageal-pressure signal. The main findings and the novelty of the proposed technique are: (i) the technique is fully automated and adaptive to the sampling rate, (ii) very high cardiogenic-oscillation removal performance, (iii) the proposed technique is applicable on both long and short duration esophageal-pressure signal signals, (iv) it does not require an adaptation time, and (v) the proposed technique is data-driven, and its performance does not depend on any pre-assumed mathematical function/model unlike the wavelet transform-based ones.

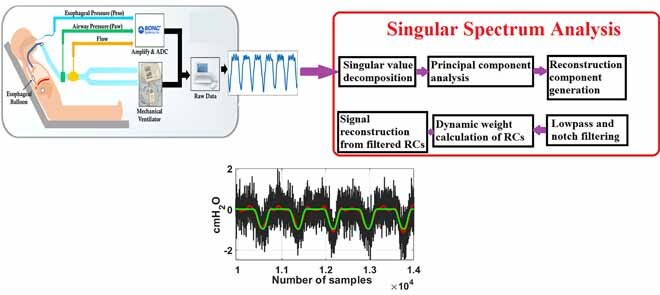

## I. Introduction

Pleural pressure is the pressure generated surrounding the lung within the pleural space during respiration. The pleural pressure well reflects the mechanics of respiration and also the work done by the respiratory muscles. However, the pleural pressure is not uniformly distributed throughout the thoracic cavity, and hence, the measured pleural pressure from a single site in the pleural space may not portray the holistic-activity of the respiratory system. Moreover, measurement of the pleural pressure from multiple sites is impractical as it is very difficult to get direct access to the pleural space, and also the process entails great risk, which might result in a collapsed lung [1]. In 1949, Buytendijk showed in his Ph.D. thesis [2] that the esophageal pressure ($\boldsymbol {P}_{ \boldsymbol {eso}}$) signal can be used as a surrogate of pleural pressure. $\boldsymbol {P}_{ \boldsymbol {eso}}$ is measured using an esophageal balloon attached to a long and thin catheter, which is placed at the lower two-thirds of the intra-thoracic esophagus. The balloon is filled with an optimum volume of air [3].

The $\boldsymbol {P}_{ \boldsymbol {eso}}$ signal often gets highly contaminated with the oscillatory-pressure-signal of the heart, which is known as the cardiogenic oscillation ($\boldsymbol {CGO}$) signal. The task of removal of the $\boldsymbol {CGO}$ from $\boldsymbol {P}_{ \boldsymbol {eso}}$ is very challenging, as the bandwidths of these two signals are very close. The bandwidth of the $\boldsymbol {P}_{ \boldsymbol {eso}}$ signal varies from 0.17 Hz to 0.67 Hz [4], and the bandwidth of the $\boldsymbol {CGO}$, i.e., the heart-rate signal varies from 0.8 Hz (48 heart-beats per minute) to 4 Hz (240 heart-beats per minute) [5]. Since the upper-band-limit of the ${ \boldsymbol {P}}_{ \boldsymbol {eso}}$ signal (0.67 Hz) and the lower-band-limit of the $\boldsymbol {CGO}$ signal (0.8 Hz) are very close (~0.13 Hz), the direct use of the conventional filters such as bandpass and band-stop filters, having fixed cut-off frequencies on $\boldsymbol {CGO}$-contaminated ${ \boldsymbol {P}}_{ \boldsymbol {eso}}$ signals might not provide a good denoising performance. Use of data-driven or adaptive filtering techniques is always better choices under such circumstances.

Schuessler *et al.* have proposed an adaptive filtering-based technique for the removal of $\boldsymbol {CGO}$ from ${ \boldsymbol {P}}_{ \boldsymbol {eso}}$ signal in [6]. The technique, which is proposed in [6] requires the ${ \boldsymbol {P}}_{ \boldsymbol {eso}}$ signal as well as the electrocardiogram (ECG) signal of the same subject, and a linear dynamic filter is used to obtain an artifact-free ${ \boldsymbol {P}}_{ \boldsymbol {eso}}$ signal. An enhanced version of the technique, which is proposed in [6] is reported in [7]. In [7], the performance of the technique is tested on ${ \boldsymbol {P}}_{ \boldsymbol {eso}}$ signals collected from eight patients only. Moreover, the adaptive filters designed in [6] and [7] require: (i) 1 minute to adapt with that of the heart rate, which suggests that both the techniques are not applicable on short-duration ${ \boldsymbol {P}}_{ \boldsymbol {eso}}$ signals and (ii) consecutive 10 stable and clean respiration efforts.

In [4], a modified adaptive noise cancellation (MANC) technique is proposed by Cheng *et al.* for denoising the ${ \boldsymbol {P}}_{ \boldsymbol {eso}}$ signal. The MANC technique utilizes the noisy ${ \boldsymbol {P}}_{ \boldsymbol {eso}}$ signal, and an airflow signal as a reference to estimate the $\boldsymbol {CGO}$. Finally, the estimated $\boldsymbol {CGO}$ signal is subtracted from the noisy ${ \boldsymbol {P}}_{ \boldsymbol {eso}}$ signal. The performance of the MANC technique is tested on the ${ \boldsymbol {P}}_{ \boldsymbol {eso}}$ signals collected from Brown-Norway rats. The bandwidths of both the ${ \boldsymbol {P}}_{ \boldsymbol {eso}}$ and $\boldsymbol {CGO}$ signals are high for rats compared to that of human. Therefore, it is difficult to premise the performance of the MANC technique on ${ \boldsymbol {P}}_{ \boldsymbol {eso}}$ signals collected from intensive care unit patients. An enhanced version of the MANC technique is reported in [8].

A template subtraction-based technique is proposed by Grabhoff *et al.* in [9] for the removal of $\boldsymbol {CGO}$ from ${ \boldsymbol {P}}_{ \boldsymbol {eso}}$ signal. Here also, in [9], a reference-signal, namely electromyogram (EMG) is required to generate a template of the $\boldsymbol {CGO}$ signal. The techniqueis designed based on three main steps: detection of the R-peak-indices from EMG signal, template generation and template subtraction.

Zara *et al.* have recently proposed an ensemble empirical mode decomposition (EEMD)-based technique for the removal of $\boldsymbol {CGO}$ from ${ \boldsymbol {P}}_{ \boldsymbol {eso}}$ signal in [10], where a reference signal is not required. In [10], the $\boldsymbol {CGO}$ contaminated ${ \boldsymbol {P}}_{ \boldsymbol {eso}}$ signal is decomposed into a number of intrinsic mode functions (IMFs), and the IMFs which are associated with the $\boldsymbol {P}_{ \boldsymbol {eso}}$ signal only, are summed up to obtain a $\boldsymbol {CGO}$-free ${ \boldsymbol {P}}_{ \boldsymbol {eso}}$ signal. However,

All the above discussed techniques [4], [6]–[9] (except [10]), require an additional signal, be it an ECG or EMG, along with ${ \boldsymbol {P}}_{ \boldsymbol {eso}}$. Acquisition of an extra signal undoubtedly increases the complexity of the system and hinders patients’ comfort. In [10], the selection of IMFs is done relying on visual inspection only, and therefore, the technique cannot be considered as a fully-automated one. Moreover, both EMD, and EEMD-based techniques are known to be high time-consuming.

Singular spectrum analysis (SSA) is a model-free and data-driven time-series-decomposition method. It decomposes a time series into three components: trend, seasonal components and noise [27]–[29]. Use of SSA-based methods have been proven to be very efficient in a wide range of applications including climatology [30], electricity consumption forecasting [31], biomedical image and signal processing [14], [14]–[33], gait parameter estimation [12]. However, the potential of denoising $\boldsymbol {CGO}$ contaminated ${ \boldsymbol {P}}_{ \boldsymbol {eso}}$ signal using a SSA-based method is not explored to date.

The motivation behind the proposed research work is to design a high-performance data-driven $\boldsymbol {CGO}$ removal technique from ${ \boldsymbol {P}}_{ \boldsymbol {eso}}$ signal addressing the hurdles, shortcomings and drawbacks of the aforementioned techniques. The main findings and the novelty of the proposed technique are: (i) it does not require an additional signal as reference, (ii) the proposed technique is fully automated and adaptive, (iii) very high $\boldsymbol {CGO}$-removal performance, (iv) the proposed technique is applicable on both long and short duration signals, (v) it does not require an adaptation time (vi) the technique is adaptive to the sampling rate of the $\boldsymbol {P}_{ \boldsymbol {eso}}$ signal, and (vii) the proposed technique is data-driven, and its performance does not depend on any pre-assumed mathematical function/model unlike the wavelet transform-based ones.

The remainder of this paper is organized as follows. The proposed singular spectrum analysis (SSA)-based data-driven ${ \boldsymbol {P}}_{ \boldsymbol {eso}}$ signal denoising technique is presented in Section II. The performance of the proposed technique is analyzed in Section III, and finally, the technique is discussed and conclusions drawn in Section IV.

## II. SSA-Based ${ \boldsymbol {P}}_{ \boldsymbol {eso}}$ Signal Denoising Technique

**FIGURE 1.**
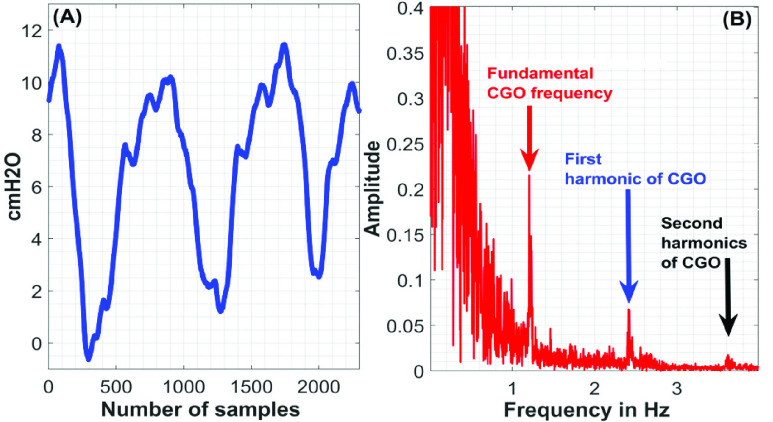
(A) $CGO$-contaminated ${P}_{eso}$ signal and (B) its DFT.

First, the discrete Fourier transform (DFT) of the $\boldsymbol {CGO}$-contaminated ${ \boldsymbol {P}}_{ \boldsymbol {eso}}$ signal is computed. In DFT-domain, the maximum peak-value, which appears at or above 0.8 Hz, is identified, and the corresponding frequency-index, which is denoted as ${ \boldsymbol {F}}_{ \boldsymbol {CGO}}$, is considered as the fundamental-frequency of the $\boldsymbol {CGO}$ signal. Figure 1 exemplifies the operation. Next, the $\boldsymbol {CGO}$-contaminated ${ \boldsymbol {P}}_{ \boldsymbol {eso}}$ signal (which is here denoted as $\boldsymbol {P}$) of length $L$ is used to form $N$-lagged vectors each of length $M$. The lagged-vectors are arranged in the form of a trajectory matrix, which is denoted as $\boldsymbol {P}_{Tra}$. The first column of the matrix $\boldsymbol {P}_{Tra}$ contains $\boldsymbol {P}$; the second column of the matrix $\boldsymbol {P}_{Tra}$ contains $\boldsymbol {P}$ shifted by 1 samples, and likewise, the $\boldsymbol {N}^{th}$ column contains $\boldsymbol {P}$ shifted by $N-1$ number of samples.

\begin{align*} \boldsymbol {P}_{Tra}=\left [{ {\begin{array}{cccccccccccccccccccc} \boldsymbol {P}_{1} &\quad \boldsymbol {P}_{2} &\quad \ldots &\quad \boldsymbol {P}_{N}\\ \boldsymbol {P}_{2} &\quad \boldsymbol {P}_{3} &\quad \ldots &\quad \boldsymbol {E}_{N+1}\\ \vdots &\quad \vdots &\quad \cdots &\quad \vdots \\ \boldsymbol {P}_{M} &\quad \boldsymbol {P}_{M+1} &\quad \ldots &\quad \boldsymbol {E}_{N+M-1} \end{array}} }\right]\tag{1}\end{align*}

where, $M=L-N+1$.

Since the elements of the anti-diagonals of $\boldsymbol {P}_{Tra}$ are the same, i.e., ${ \boldsymbol {P}}_{ij}= \boldsymbol {P}_{ji}(i\ne j)$, the trajectory matrix $\boldsymbol {P}_{Tra}$ is a Henkel matrix. In the SSA operation, the selection of the proper value of the window-length $N$ is of great importance. Precise separation of two signals from a composite one is possible using SSA technique if their frequency-difference is ${\ge } \frac {M}{N}\times \frac {1}{M}=\frac {1}{N}Hz$. On the other hand, SSA fails to separate two signals from a composite signal if (1) the amplitudes of both the signals are equal, or (2) their frequency-difference is even less than $\frac {1}{N}Hz$. A detail theoretical-explanation and mathematical-exploration of SSA method can be found in [18]. The value of $N$ should be chosen in such a way that all the respiratory-efforts, i.e., the inspiratory and expiratory processes, which are present in $\boldsymbol {P}$, are also to be present in each of the lagged vectors. A small value of $N$ would not help in denoising the signal. On the other hand, a large value of $N$ is not only redundant, but it also increases the computational burden. Most importantly, a large value of $N$ may cause overlapping of the respiratory events, which in turn leads to a significant loss of the clinical information. In this research work, the window-length $N$ is determined based on the inherent periodicity and morphological properties of the inhalation and exhalation events. The maximum rate of inhalation for humans, which is reported in the literature [4], is 40 breaths per minute. Thus, the window-length $N$ is calculated as shown in Equation 2.

\begin{equation*} N=\frac {60\times sampling~rate~(Hz)}{40}\tag{2}\end{equation*}

From Equation 2 it can be noted that (i) all the respiratory-efforts which are present in $\boldsymbol {P}$, are guaranteed to be present in all the columns of $\boldsymbol {P}_{Tra}$, and (ii) none of the respiratory-efforts, which are present in the adjacent columns of the $\boldsymbol {P}_{Tra}$ would overlap with its preceding respiratory-effort.

Next, the matrix $\boldsymbol {P}_{Tra}$ is decomposed using SVD to represent it as a sum of rank-one biorthogonal elementary matrices. The SVD technique factorizes a matrix into the product of another three matrices: an orthogonal matrix ($\boldsymbol {U}$), a diagonal matrix ($\boldsymbol {S}$) and the transpose of an orthogonal matrix (${V}$) [11]. Eigenvalues of the matrix $\boldsymbol {S}= \boldsymbol {P}_{ \boldsymbol {Tra}}^{ \boldsymbol {T}}\times \boldsymbol {P}_{ \boldsymbol {Tra}}$ are denoted as $\lambda =\lambda _{1},\lambda _{2},\ldots,\lambda _{N}$. The Eigenvalues are in decreasing order of magnitude (i.e., ${\lambda }_{1}\ge \lambda _{2}\ge \cdots \ge \lambda _{N}$). The corresponding eigenvectors of $\boldsymbol {S}$ are denoted as $\boldsymbol {U}={[u}_{1},u_{2},\ldots,u_{M}]$. If ${v}_{i}=P_{Tra}^{T}u_{i}/\sqrt {\lambda _{i}} $, then it is also possible to write the trajectory matrix as:

\begin{equation*} P_{Tra}=\sum \limits _{i=1}^{d} {\sqrt {\lambda }_{i} u_{i}v_{i}^{T}} {=P}_{Tra}^{1}+P_{Tra}^{2}+\cdots. P_{Tra}^{d}\tag{3a}\end{equation*}

where $d={argmax}_{i}\{\lambda _{i}>0\}$, $P_{Tra}^{i}=\sqrt {\lambda _{i}} u_{i}v_{i}^{T},u_{i}$ is the $i^{th}$ left eigenvector, and $v_{i}$ is the $i^{th}$ right eigenvector [12]. In the SVD operation, the matrix $V({=[v}_{1}, v_{2},\ldots,v_{L}])$ is denoted as the right singular matrix, and it contains the information about the most important axis of the data. The vector $v_{1}$ reveals the direction having the most variance, and $v_{L}$ reveals the direction having the least variance. The singular values define the variance precisely. The strong inter-sample and inter-cycle correlations that exist in the respiratory signal help enhancing the covariance among the eigenvector of the $\boldsymbol {U}$ matrix. The SVD operation arranges the singular values in its decreasing order of magnitude, and the small singular values barely carry any signal-information. Therefore the small singular values could be discarded [13]. $\boldsymbol {P}$ can be considered as the composition of the ${ \boldsymbol {P}}_{ \boldsymbol {eso}}$ signal and the $\boldsymbol {CGO}$. If the indices $I=\{i_{1},\ldots i_{p}\}(p < d)$ of the eigenvalues, which hold the most of the ${ \boldsymbol {P}}_{ \boldsymbol {eso}}$ signal-information, are known, then the matrix corresponding to the ${ \boldsymbol {P}}_{ \boldsymbol {eso}}$-signal-only can be written as follows.

\begin{align*} \boldsymbol {P}_{ \boldsymbol {P}_{ \boldsymbol {eso}}}=\sum \limits _{i=i_{1}}^{i_{p}} P_{Tra}^{i} =\left [{ {\begin{array}{cccccccccccccccccccc} \overline { \boldsymbol {P}_{1,1}} &\quad \overline { \boldsymbol {P}_{1,2}} &\quad \ldots &\quad \overline { \boldsymbol {P}_{1,M}}\\ \overline { \boldsymbol {P}_{2,1}} &\quad \overline { \boldsymbol {P}_{2,2}} &\quad \ldots &\quad \overline { \boldsymbol {P}_{2,M}}\\ \vdots &\quad \vdots &\quad \cdots &\quad \vdots \\ \overline { \boldsymbol {P}_{N,1}} &\quad \overline { \boldsymbol {P}_{N,2}} &\quad \ldots &\quad \overline { \boldsymbol {P}_{N,M}} \end{array}} }\right]\tag{3b}\end{align*}

The principal components $(\boldsymbol {PC}s)$ of $\boldsymbol {P}$ are computed by a linear combination of the matrix $\boldsymbol {P}_{Tra}$ and the matrix of eigenvectors $\boldsymbol {V}$.

\begin{equation*} { \boldsymbol {PC}}_{M\times N}= \boldsymbol {P}_{Tra}^{T}\times \boldsymbol {V}\tag{4a}\end{equation*}

The columns of the matrix $\boldsymbol {PC}$ are the principal components of $\boldsymbol {P}$. Next, a matrix is created by an inverse projection of the principal components and the matrix of eigenvectors $\boldsymbol {V}$. Finally, the reconstructed components ($\boldsymbol {RC}\text{s}$) of the original ${ \boldsymbol {P}}_{ \boldsymbol {eso}}$ signal are computed by averaging along the anti-diagonals of this matrix [14].

\begin{equation*} {RC}_{i}=\frac {1}{R(W_{i})}\sum \limits _{(l,k)\in A_{i}} {\overline { \boldsymbol {P}_{l,k}}}\tag{4b}\end{equation*}

where, $W_{i}=\{\left ({l,k }\right):l+k=i+1,1\le l\le N,1\le k\le M\}$, $R(W_{i})$ is the number of elements in $W_{i}$. The original ${ \boldsymbol {P}}_{ \boldsymbol {eso}}$ signal $\boldsymbol {P}$ can be obtained by summing up all the ${RC}_{s}$.

\begin{equation*} \boldsymbol {P}=\sum \limits _{ \boldsymbol {i}= \boldsymbol {1}}^{ \boldsymbol {N}} {RC_{i}}\tag{4c}\end{equation*}

**FIGURE 2.**
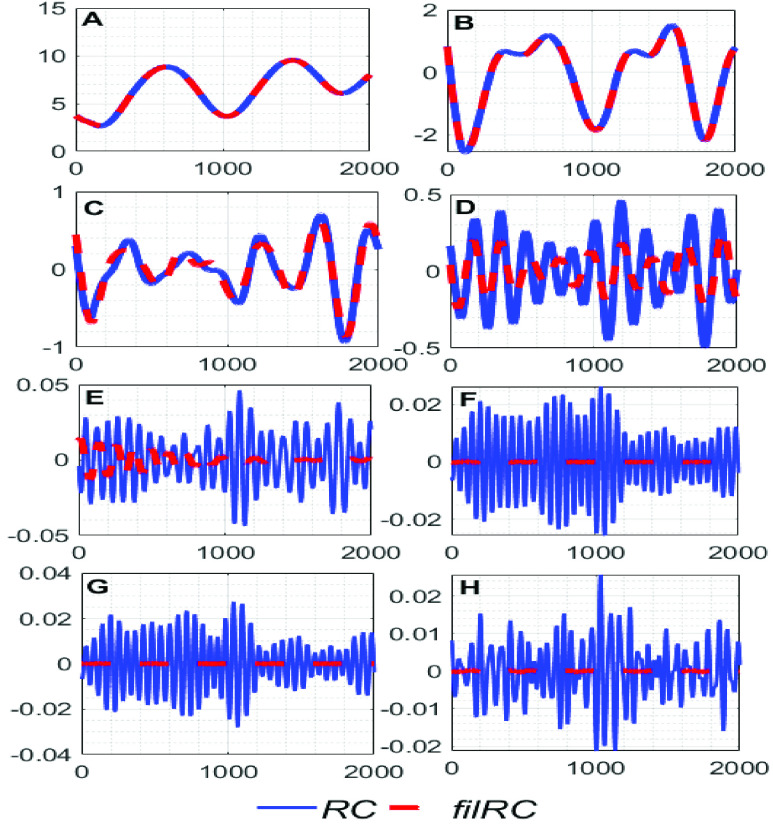
(A) First, (B) second, (C) third, (D) forth, (E) $10^{\mathrm {th}}$, (F) $11^{\mathrm {th}}$, (G) $12^{\mathrm {th}}$, (H) $13^{\mathrm {th}}~{\textit {RCs}}$ and ${\textit {filRCs}}$. The units of the X and Y axes of figures A-H are the number of samples and cmH2O, respectively.

**FIGURE 3.**
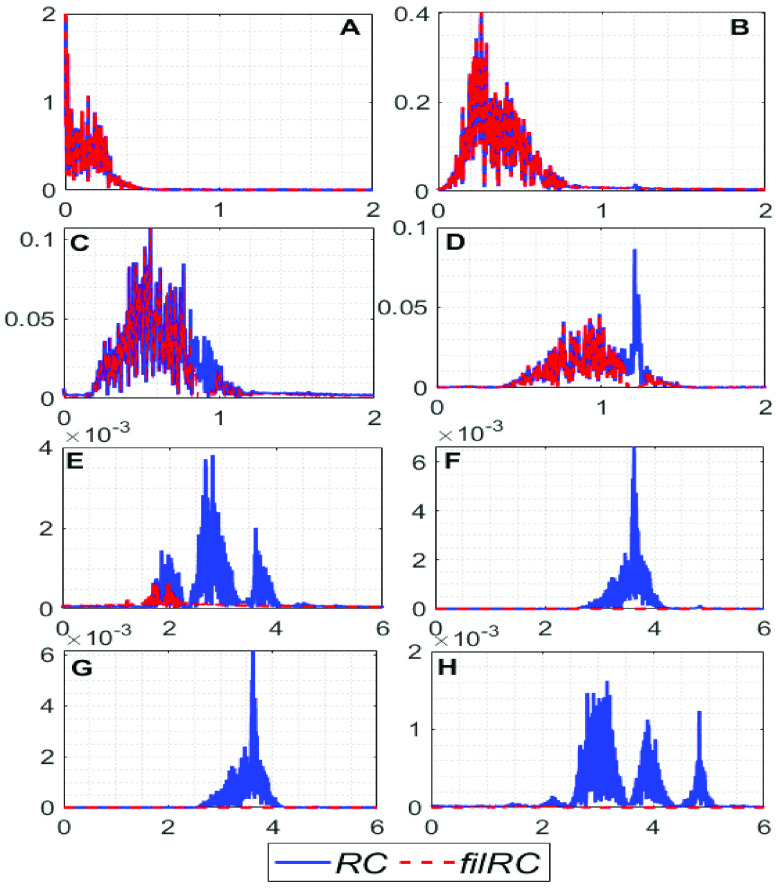
Frequency spectrum of the (A) First, (B) second, (C) third, (D) forth, (E) $10^{\mathrm {th}}$, (F) $11^{\mathrm {th}}$, (G) $12^{\mathrm {th}}$, (H) $13^{\mathrm {th}}~{\textit {RCs}}$ and ${\textit {filRCs}}$. The units of the X and Y axes of figures A-H are frequency in hertz and amplitude, respectively.

Now, all the $\boldsymbol {RC}\text{s}$ are filtered using a zero-phase $4^{\mathrm {th}}$ order Butterworth bandpass filter having lower and upper cut-off frequencies 0.17 Hz and 1.4 Hz, respectively, and a zero-phase $4^{\mathrm {th}}$ order Butterworth notch filter, whose center-frequency is set to ${ \boldsymbol {F}}_{ \boldsymbol {CGO}}$ in order to remove the interference of the $\boldsymbol {CGO}$ signal. The filtered $\boldsymbol {RC}\text{s}$ are denoted as $\boldsymbol {filRC}s$. Figure 2 shows an example of a few $\boldsymbol {RC}\text{s}$ and $\boldsymbol {filRC}s$, and Figure 3 shows their corresponding frequency-spectras. From Figure 2 it can be observed that the low-order $\boldsymbol {RC}\text{s}$ remain almost unaltered, and the high-order $\boldsymbol {RC}\text{s}$ are almost diminished after the filtering operation, which suggests that the low-order $\boldsymbol {RC}\text{s}$ are signal dominant, and the high-order $\boldsymbol {RC}\text{s}$ are noise dominant. Figure 3 corroborates this notion.

A dynamic weight calculation-based method is used to discard the less-significant high-order $\boldsymbol {filRC}\text{s}$. The weights of all the $\boldsymbol {filRC}\text{s}$ are calculated as follow:

\begin{align*}&\hspace {-0.5pc}W\left ({n }\right)=\frac {\sum _{i=1}^{M} {{ \boldsymbol {filRC}}_{n}^{2}\left ({i }\right)}}{\sum _{n=1}^{N} \sum _{i=1}^{M} {{ \boldsymbol {filRC}}_{n}^{2}\left ({i }\right)}}\times 100\% \\&\qquad \qquad\qquad\qquad\qquad {{\displaystyle {n=1,2,3,\ldots.,N} }}\tag{5}\end{align*}

where $W\left ({n }\right)$ is the weight of the ${n}^{th}~ \boldsymbol {filRC}$, $N$ denotes the total number of $\boldsymbol {filRC}s$, and ${ \boldsymbol {filRC}}_{n}(i)$ denotes the $i^{th}$ coefficients of the ${n}^{th}~ \boldsymbol {filRC}$. Finally, an optimum number of $\boldsymbol {filRC}s$ are summed-up until the cumulative weight reaches a predefined threshold value. The threshold value is denoted as ${Th}_{W}$. The value of ${Th}_{W}$, which is used in this research work, is explained in Section III. The summed-up $\boldsymbol {filRC}s$ is considered as the denoised ${ \boldsymbol {P}}_{ \boldsymbol {eso}}$ signal, i.e., the $\boldsymbol {CGO}$-free ${ \boldsymbol {P}}_{ \boldsymbol {eso}}$ signal. The $\boldsymbol {filRC}$-summing algorithm is given below.

Step 1: $n=1,rec=0,weigh=0$
Step 2: $rec=rec+{ \boldsymbol {filRC}}_{n}$
Step 3: $weigh=weigh+W(n)$
Step 4: $if\,\,weigh\ge {Th}_{W}$
  $\boldsymbol {d} \boldsymbol {P}_{ \boldsymbol {eso}}=rec$
  $n_{opt}=n$
  else
  $n=n+1$
  *go to Step*2
  end

where $n_{opt}$ is the optimum number $\boldsymbol {filRC}s$ which is required to attain ${Th}_{W}$.

## III. Performance of the Proposed Technique

The performance of the proposed technique is quantified through qualitative as well as quantitative measures. The technique is implemented on MATLAB platform with a computer having 64-bit Windows 7 operating system, 16GB RAM and Intel Xeon CPU E3-1225 v3 3.20 GHz.

### A. Data Acquisition

**FIGURE 4.**
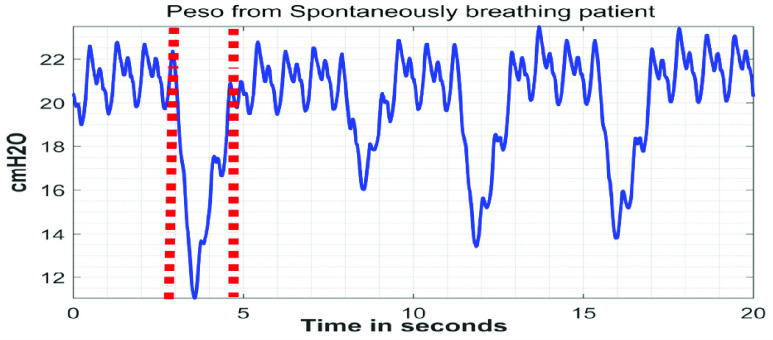
A ${P}_{eso}$ signal from a spontaneously breathing patient. The red vertical dashed lines represent the duration of one inspiratory cycle. At the onset of inspiration, the respiratory muscles contract resulting in the negative deflection of the $P_{eso}$ signal.

**FIGURE 5.**
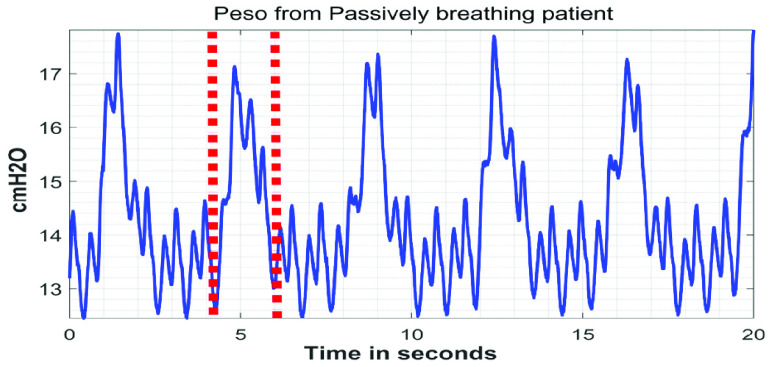
A $P_{eso}$ signal from a passively breathing patient. The red vertical dashed lines represent the duration of one inspiratory cycle. At the onset of inspiration, the mechanical ventilator provides positive pressure to allow air flow into the lungs resulting in the positive deflection of the $P_{eso}$ signal.

The ${ \boldsymbol {P}}_{ \boldsymbol {eso}}$ signals are collected in three different settings as shown in Table 1. Under Setting #1 in Table 1, 75 signals are acquired from 25 patients being monitored in the ICU at St. Michael’s Hospital in Toronto, Ontario, Canada. The collection of this data is approved by St. Michael’s Hospital’s Research Ethics Board. For each of the 25 patients, signals are captured under three conditions: i) spontaneous breathing, ii) breathing using a t-piece, and iii) passive breathing. T-piece is a T-shape tubing that can be connected to the endotracheal tube while the patient is disconnected from the ventilator allowing for oxygen supplementation normally used during the so called “spontaneous breathing trials” to test the patient’s ability to breathe without the ventilator’s assistance prior to removal of the endotracheal tube. Spontaneous breathing and breathing using a T-piece are considered to be ‘active breathing’ conditions, which signifies that the patients are able to recruit their respiratory muscles to provide some degree of effort during inspiration. In this dataset, the spontaneously breathing patients received pressure support ventilation (PSV). During PSV, the patients’ inspiratory efforts triggered the ventilator to provide a pre-set positive pressure to supplement the patients’ breath. On the other hand, when the patients were breathing using a T-piece, they were provided an external oxygen supply but were temporarily disconnected from the mechanical ventilator. The use of PSV and/or T-piece are common methods of weaning patients off mechanical ventilation by encouraging them to take on a greater proportion of the ventilatory effort. The other condition in this dataset is ‘passive breathing’, which means that the patients did not provide any inspiratory effort during inspiration. Under this condition, respiration is achieved solely by the mechanical ventilator. To achieve the passive breathing condition in this dataset, the patients were sedated, and a paralysis-inducing drug was administered to their respiratory muscles. The distinction between ‘active’ and ‘passive’ breathing conditions is important, because the morphology of the $\boldsymbol {P}_{ \boldsymbol {eso}}$ signal changes depending on the condition. During ‘active’ breathing, the $\boldsymbol {P}_{ \boldsymbol {eso}}$ signal will exhibit a negative deflection during inspiration. The reason for the negative deflection is because during inspiration, the respiratory muscles contract and the volume of the chest cavity increases. An increase in the chest cavity volume generates a pressure gradient and allows for air to flow into the lungs. Since pressure is inversely proportional to volume according to Boyle’s Law [19], a negative deflection in the $\boldsymbol {P}_{ \boldsymbol {eso}}$ signal is observed. Figure 4 illustrates an example of a $\boldsymbol {P}_{ \boldsymbol {eso}}$ signal from a spontaneously breathing patient. In passive-breathing conditions, the patients’ respiratory muscles do not contract during inspiration. Thus, the $\boldsymbol {P}_{ \boldsymbol {eso}}$ signal will deflect upwards during inspiration as a result of mechanical insufflations and the chest-wall elastance. Figure 5 shows an example of a $\boldsymbol {P}_{ \boldsymbol {eso}}$ signal from a passively-breathing patient.

**TABLE 1:** Details of the Collected $P_{eso}$ Signals

Settings #	Where?	From whom?	Duration of each signal (seconds)	No. of signal acquired	Sampling rate
1	ICU, SMH	Patients	50.92 - 644.86	75	200 Hz
2	SMH	Synthetic	55 – 304.8	9	
3	Cadaver laboratory, UQTR [17]	Deceased bodies	429.91 – 524.28	2	

UQTR$\to $University of Quebec at Trois-Rivieres, SMH$\to $St. Michael’s Hospital

Setting #2 represents synthetic data under an ‘active’ breathing condition. Nine $\boldsymbol {P}_{ \boldsymbol {eso}}$ signals were simulated using the IngMar Medical ASL 5000 (Active Servo Lung) High Fidelity Breathing Simulator and are normalized to ±1. The settings that are used on ASL5000 to generate the nine simulated $\boldsymbol {P}_{ \boldsymbol {eso}}$ signals are shown in Table 2.

**TABLE 2:** Simulator Settings Used to Generate Simulated $P_{eso}$ Signals

$\boldsymbol {P}_{ \boldsymbol {eso}}$ signal #	Respiratory Rate (breaths /min)	$\boldsymbol {P}_{ \boldsymbol {eso}}$ Swing (cmH2O)	Functional Residual Capacity (mL)	Compliance (mL /cmH2O)	Resistance (cmH2O /mL)
1	15	−10	500	20	10
2	17	−10	500	20	10
3	19	−10	500	20	10
4	21	−10	500	20	10
5	23	−10	500	20	10
6	27	−10	500	20	10
7	29	−10	500	20	10
8	31	−10	500	20	10
9	15	−5 to −15	500	20	10

The last set of data, under setting #3, was captured during cadaver studies. The $\boldsymbol {P}_{ \boldsymbol {eso}}$ signals captured from cadavers takes into account the complex characteristics of the chest wall, respiratory muscles, and the pulmonary system during inspiration and expiration. The cadaver $\boldsymbol {P}_{ \boldsymbol {eso}}$ signals provided another approach to testing and validating the proposed technique since no $\boldsymbol {CGO}$ is present in the signal.

Two types of catheters were used in the clinical study and in the cadavers: i) Adult Esophageal Balloon Catheter (Cooper Surgical, Inc., USA) consisting of 86 cm closed-end catheter with multiple perforations surrounded by a balloon of 9.5 cm length (polyethylene) [20] and ii) Nutrivent Multifunction Naso-Gastric Catheter (Sidam, Italy) consisting of 108 cm closed-end catheter with multiple perforations surrounded by a balloon of 10 cm length (polyethylene) [21].

### B. Qualitative Measure

First, all the ${ \boldsymbol {P}}_{ \boldsymbol {eso}}$ signals, which are collected in setting #1, are denoised using the proposed technique, and the performance of the technique is assessed through qualitative assessment. Semi-blind mean opinion score (MOS) test [15] of thirteen clinicians (clinicians having extensive expertise and experience; 3-years to 25-years, on ${ \boldsymbol {P}}_{ \boldsymbol {eso}}$ signal analysis) from around the globe (8 different countries) were carried out. A web-based survey-form has been created containing the $\boldsymbol {CGO}$-contaminated ${ \boldsymbol {P}}_{ \boldsymbol {eso}}$ signals and the corresponding denoised ${ \boldsymbol {P}}_{ \boldsymbol {eso}}$ signals. Three ${ \boldsymbol {P}}_{ \boldsymbol {eso}}$ features: (i) end-expiratory pressure ($\boldsymbol {P}_{ \boldsymbol {ee}}$), (ii) $\boldsymbol {P}_{ \boldsymbol {eso}}$-pressure swing ($\Delta \text{P}$), (iii) pressure-changing-instance ($\boldsymbol {t}_{ \boldsymbol {PC}}$), and the overall ${ \boldsymbol {P}}_{ \boldsymbol {eso}}$ denoising performance were considered for the MOS test. The evaluators were asked to quantify whether the features in the denoised ${ \boldsymbol {P}}_{ \boldsymbol {eso}}$ signal contained sufficient clinical information for monitoring and diagnosis by providing a quality rating. The quality ratings were: 1 (does not contain any diagnostic information), 2, 3, 4, and 5 (contain sufficient diagnostic information). MOS of the denoised ${ \boldsymbol {P}}_{ \boldsymbol {eso}}$ signals were calculated using the following equation.

\begin{equation*} MOS=\frac {1}{N_{eva}N_{feat}}\sum \limits _{e=1}^{N_{eva}} \sum \limits _{f=1}^{N_{feat}} {Q\left ({e,f }\right)}\tag{6}\end{equation*}

where $N_{eva} =$ total number of evaluators, $N_{feat} =$ total number of features, $Q =$ quality rating of the $f^{th}$ feature given by the $e^{th}$ evaluator.

The MOS value of a particular ${ \boldsymbol {P}}_{ \boldsymbol {eso}}$ feature is expressed as

\begin{equation*} MOS\left ({f }\right)=\frac {1}{N_{eva}}\sum \limits _{e=1}^{N_{e}} {Q\left ({e,f }\right)}\tag{7}\end{equation*}

MOS error of each feature and the overall denoised ${ \boldsymbol {P}}_{ \boldsymbol {eso}}$ signal are calculated using Equation 8 and 9, respectively.

\begin{align*} {MOS}_{f}=&\left ({1-\frac {MOS\left ({f }\right)}{5} }\right)\times 100\% \tag{8}\\ {MOS}_{e}=&\left ({1-\frac {MOS}{5} }\right)\times 100\%\tag{9}\end{align*}

According to the $MOS$ error criteria, the quality of the reconstructed biosignal is considered to be (i) ‘very good’ if the $MOS$ error lies in between 0 and 15%, and (ii) ‘good’ if it lies in between 15% and 35% [16]. Table 3 shows the $MOS$ errors of various $\boldsymbol {P}_{ \boldsymbol {eso}}$ features and overall denoised ${ \boldsymbol {P}}_{ \boldsymbol {eso}}$ signals. According to MOS error criteria the reconstructed ${ \boldsymbol {P}}_{ \boldsymbol {eso}}$ signals (overall) and also all of its features fall under the ‘very good’ category.

**TABLE 3:** MOS Error of Various Feature and Overall $P_{eso}$ Signals

Features	MOS error	
	${MOS}_{f}$ (%)	${MOS}_{e}$ (%)
$P_{ee}$	9.67	10.42
$\Delta \text{P}$	11.17	
$t_{PC}$	9.67	
Overall denoising performance	11.17	

**FIGURE 6.**
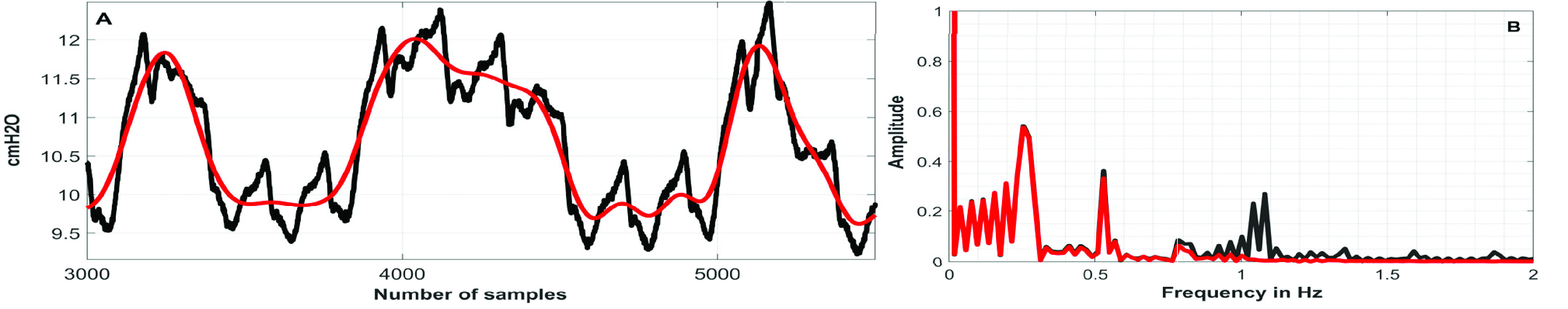
(A) Black$\to {CGO}$-contaminated $P_{eso}$ signal, red$\to $denoised $P_{eso}$ signals, (B) Black$\to $Frequency spectra of $CGO$-contaminated $P_{eso}$ signal, red$\to $Frequency spectra of denoised $P_{eso}$ signals.

**FIGURE 7.**
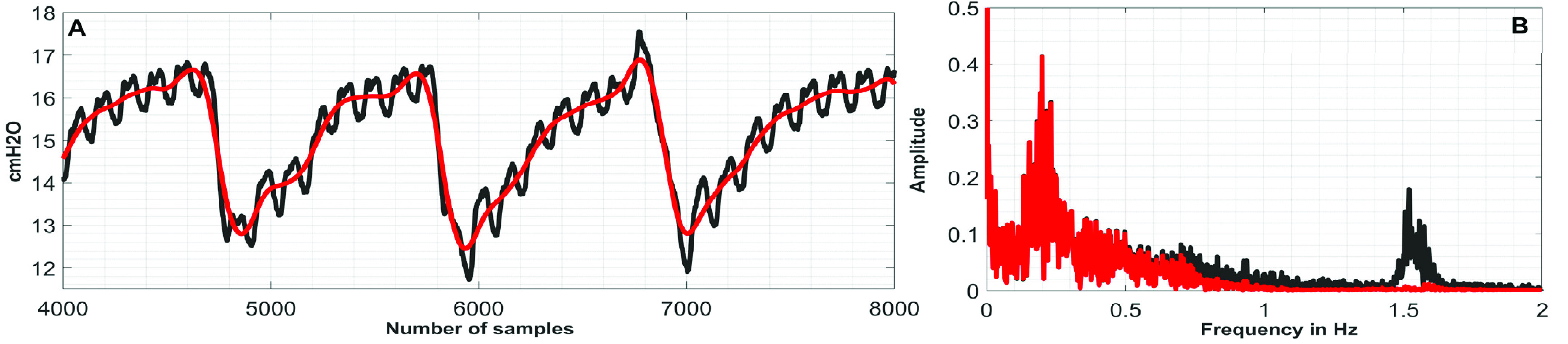
(A) Black$\to CGO$-contaminated $P_{eso}$ signal, red$\to $denoised $P_{eso}$ signals, (B) Black$\to $Frequency spectra of $CGO$-contaminated $P_{eso}$ signal, red$\to $Frequency spectra of denoised $P_{eso}$ signals.

**FIGURE 8.**
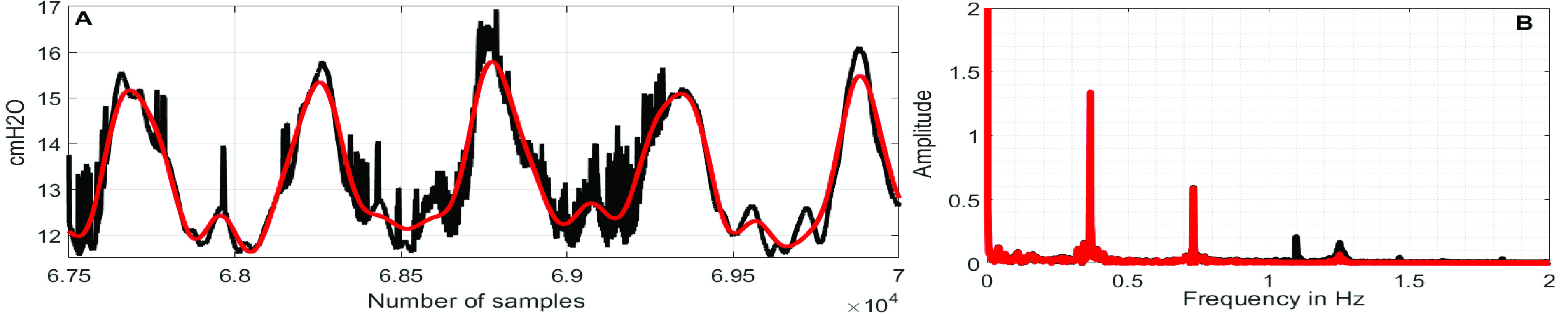
(A) Black$\to CGO$-contaminated $P_{eso}$ signal, red$\to $denoised $P_{eso}$ signals, (B) Black$\to $Frequency spectra of $CGO$-contaminated $P_{eso}$ signal, red$\to $Frequency spectra of denoised $P_{eso}$ signals.

Figures 6 to 8 show the $\boldsymbol {CGO}$ removal efficiency of the proposed technique on ${ \boldsymbol {P}}_{ \boldsymbol {eso}}$ signals, which are collected in setting #1. Here it is worth mentioning that the minimum heart rate, which has been encountered while denoising the 75 signals collected in setting #1 is 53 beats per minute (~0.88 Hz).

### C. Quantitative Measure

In doing the quantitative assessment, the $\boldsymbol {CGO}$ signals are extracted from all $75~{ \boldsymbol {P}}_{ \boldsymbol {eso}}$ signals (which are collected in setting #1) using Equation 10.

\begin{align*}&\hspace {-0.5pc} \boldsymbol {CGO}=CGO~contaminated~ \boldsymbol {P}_{ \boldsymbol {eso}}~signal \\&\qquad \qquad\qquad\qquad\qquad\qquad {{\displaystyle {-denoised\,\, \boldsymbol {P}_{ \boldsymbol {eso}}\,\,signal} }}\tag{10}\end{align*}

Next, the extracted $\boldsymbol {CGO}$ signals are added with the ${ \boldsymbol {P}}_{ \boldsymbol {eso}}$ signals that are acquired in settings #2 and #3, using Equation 11.

\begin{equation*} Syn \boldsymbol {P}_{ \boldsymbol {eso}}= \boldsymbol {P}_{ \boldsymbol {eso}}^{\mathbf {2,3}}+\overline { \boldsymbol {CGO}}\times (\mathrm {max}\vert \boldsymbol {P}_{ \boldsymbol {eso}}^{\mathbf {2,3}}\vert \times F)\tag{11}\end{equation*}

where, $\overline { \boldsymbol {CGO}}=\frac {CGO}{\mathrm {max}\vert CGO\vert }$, $F$ is the scaling factor, which varies from 0.1 to 1, and $\boldsymbol {P}_{ \boldsymbol {eso}}^{\mathbf {2,3}}$ is the ${ \boldsymbol {P}}_{ \boldsymbol {eso}}$ signal collected in settings #2 and #3.

In total, $1800~Syn \boldsymbol {P}_{ \boldsymbol {eso}}$ signals are generated using Equation 11. The $Syn \boldsymbol {P}_{ \boldsymbol {eso}}$ signals are then denoised using the proposed technique. Next, the quality of the denoised-$Syn \boldsymbol {P}_{ \boldsymbol {eso}}$ signals are quantified through quantitative measures, such as percent root mean square ($PRD$). $PRD$ is a widely accepted numerical measure for assessing the quality or acceptability of processed biosignals [15]. Hence, $PRD$ is used in this research work to gauge the quality of the denoised $Syn \boldsymbol {P}_{ \boldsymbol {eso}}$ signal. The $PRD$ values are calculated using Equation 12.

\begin{equation*} PRD\left ({\% }\right)=\sqrt {\frac {\sum _{i=1}^{N} {(\boldsymbol {P}_{ \boldsymbol {eso}}^{\mathbf {2,3}}\left ({i }\right)-\overline {Syn \boldsymbol {P}_{eso}(i))}}^{2}}{\sum _{i=1}^{N} {(\boldsymbol {P}_{ \boldsymbol {eso}}^{\mathbf {2,3}}\left ({i }\right))}^{2}}} \times 100\tag{12}\end{equation*}

where, and $\overline {Syn \boldsymbol {P}_{eso}}$ is the denoised-$Syn \boldsymbol {P}_{ \boldsymbol {eso}}$ signal.

**FIGURE 9.**
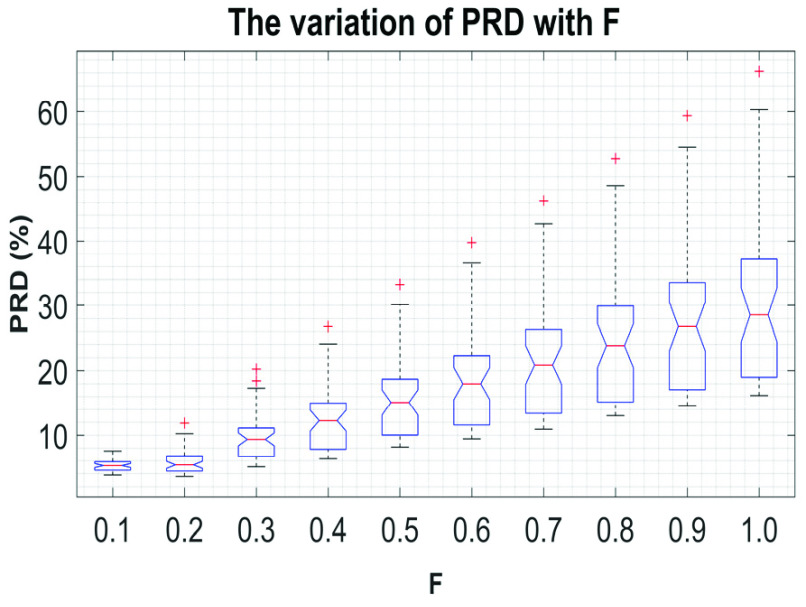
The variation of $PRD$ with $F$.

**FIGURE 10.**
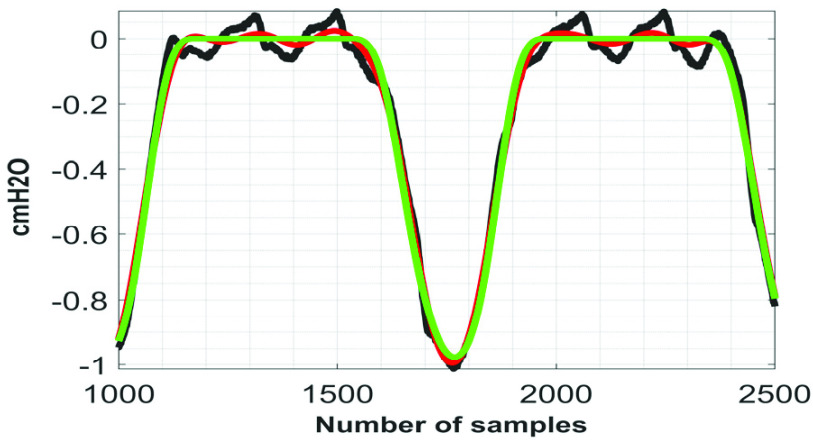
Black$\to SynP_{eso}$, red$\to \overline {SynP_{eso}}$, green$\to P_{eso}^{2,3}.\,\,F=0.1.\,\,PRD=4.62\% $.

**FIGURE 11.**
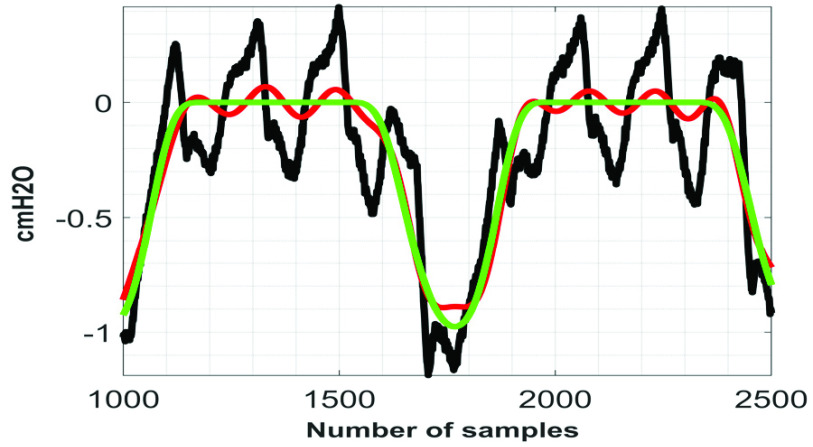
Black$\to Syn P_{eso}$, red$\to \overline {SynP_{eso}}$, green$\to P_{eso}^{2,3}$. $F=0.5$. $PRD=14.21\% $.

**FIGURE 12.**
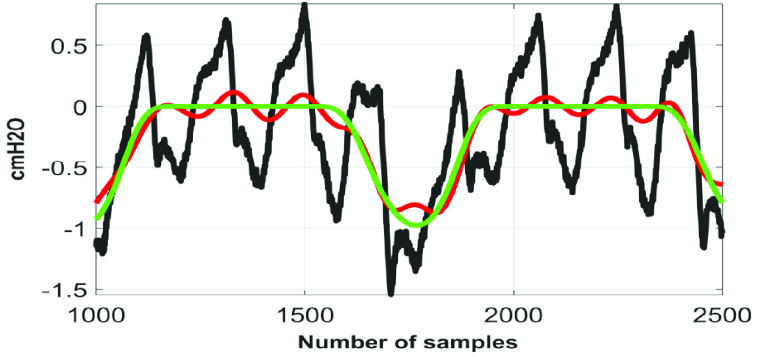
Black$\to SynP_{eso}$, red$\to \overline {SynP_{eso}}$, green$\to P_{eso}^{2,3}$. $F=1.0$. $PRD=27.07\% $.

According to the globally considered standard, the quality of the reconstructed biosignal is considered to be (i) ‘very good’ if the PRD value lies in between 0 and 2%, and (ii) ‘good’ if the PRD value lies in between 2% and 9% [16]. Figure 9 shows the variation of $PRD$ with $F$ on $Syn \boldsymbol {P}_{ \boldsymbol {eso}}$ signal. From this figure it can be noted that the quality of the reconstructed signals fall under the category ‘good’ at $F \le 0.3$. Figures 10 to 12 show the denoising performance of the proposed technique on $Syn \boldsymbol {P}_{ \boldsymbol {eso}}$ signals at different values of $F$.

**FIGURE 13.**
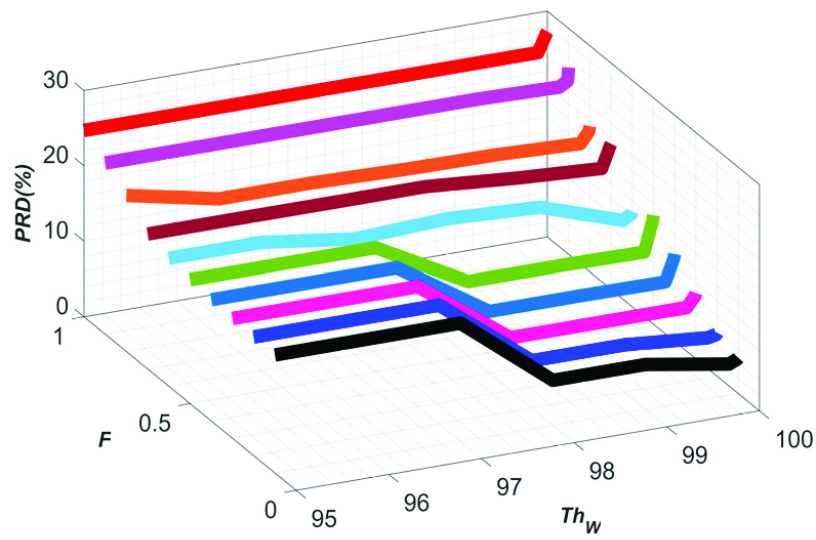
The variation of $PRD$ with $F$ and ${Th}_{W}$.

The variation of $PRD$ with $F$ and ${Th}_{W}$ is shown in Figure 13. From this figure it can be seen that the $PRD$ values are reduced with increasing ${Th}_{W}$ for all the values of $F$, and the minimum $PRD$ value is obtained at ${Th}_{W}=99.99{\%}$. As the last few $\boldsymbol {filRC}s$ are dominated by the components of the $\boldsymbol {CGO}$ signal, the $PRD$ value becomes high when all the $\boldsymbol {filRC}s$ are summed-up, i.e., at ${Th}_{W}=100{\%}$. Therefore, in this research, the value of ${Th}_{W}$ is set to 99.99%.

**FIGURE 14.**
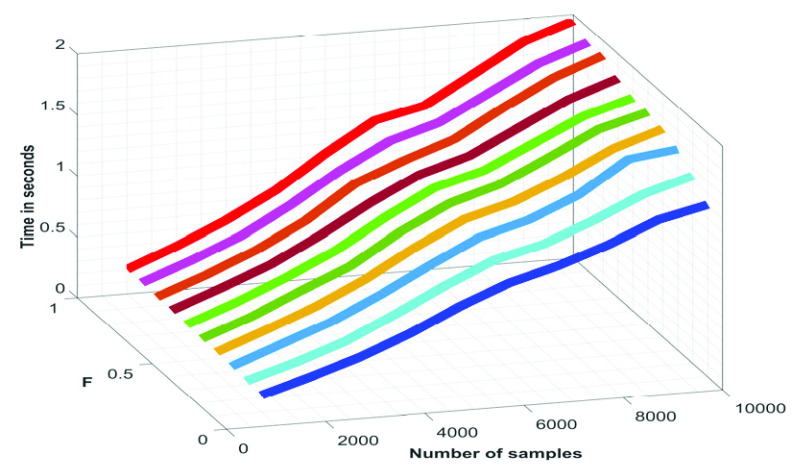
Variation in the runtime of the proposed ${P}_{eso}$ signal denoising as a function of length of the input signal at different values of $F$.

**FIGURE 15.**
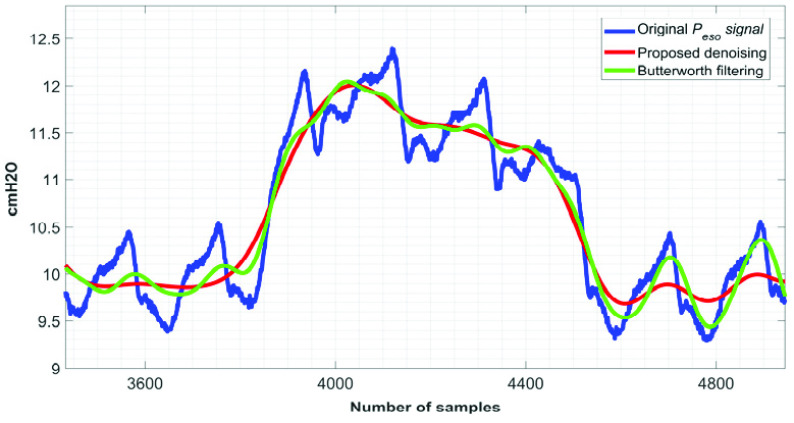
Blue$\to CGO$ contaminated ${P}_{eso}$ signal, green$\to $performance of a Butterworth bandpass and notch filter-based ${P}_{eso}$ signal denoising technique, red$\to $performance of the proposed SSA-based ${P}_{eso}$ signal denoising technique.

Time-complexity is a figure of merit, which is often used to gauge the runtime of a technique. The runtime of the proposed technique as a function of $F$ and the length of the signal is show in Figure 14. From Figure 14 it can be noted that the runtime of the proposed ${ \boldsymbol {P}}_{ \boldsymbol {eso}}$ signal denoising technique varies linearly with both the length of the input signal and $F$. In this proposed ${ \boldsymbol {P}}_{ \boldsymbol {eso}}$ signal denoising technique, the reconstructed components are filtered through Butterworth bandpass and notch filters. What would be the result if the bandpass and notch filters are directly applied on the $\boldsymbol {CGO}$ contaminated ${ \boldsymbol {P}}_{ \boldsymbol {eso}}$ signal, is shown in Figure 15. From this figure it can be noted that proposed SSA-based ${ \boldsymbol {P}}_{ \boldsymbol {eso}}$ signal denoising technique performs much better than that of when a bandpass and notch filter are used alone.

## IV. Performance Comparison

**FIGURE 16.**
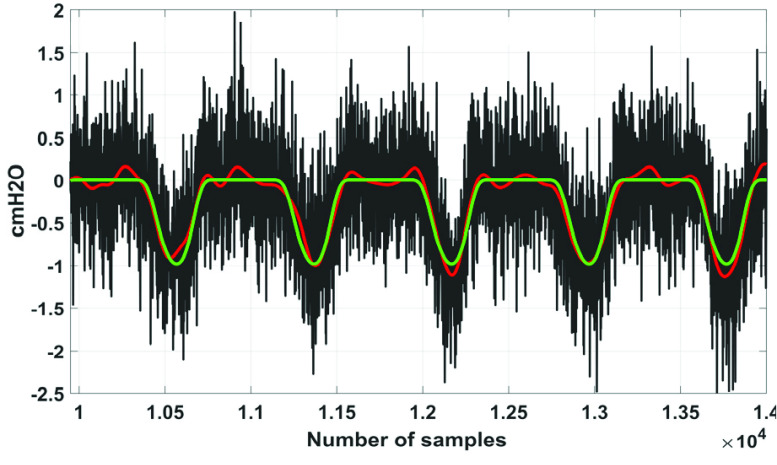
Green$\to {P}_{eso}$ signal collected in settings #2 or 3, Black$\to CGO$ ($F=0.5$) and WGN contaminated ${P}_{eso}$ signal, and Red$\to $denoised ${P}_{eso}$ signal. Input-SNR = −1.92 dB, output-SNR = −0.98 dB, PRD $=16.24$%.

A direct comparison of performance of the proposed ${ \boldsymbol {P}}_{ \boldsymbol {eso}}$ signal denoising technique with that of other techniques which are reported in [4] and [6]–[9] is not possible as the performance these techniques are evaluated with different settings and ${ \boldsymbol {P}}_{ \boldsymbol {eso}}$ databases. However, in [6], the simulated ${ \boldsymbol {P}}_{ \boldsymbol {eso}}$ signals were added with artificial $\boldsymbol {CGO}$ and white Gaussian noise (WGN) to have a signal-to-noise ratio of 10 dB, and then the noisy signals are denoised. The proposed technique is also tested likewise. A $\boldsymbol {P}_{ \boldsymbol {eso}}^{\mathbf {2,3}}$ signal is added with $\boldsymbol {CGO}$ (at an $F$ value of 0.5) and WGN (the input-SNR = −1.92 dB), and then the signal is denoised using the proposed technique. The performance of the proposed technique under the presence of both $\boldsymbol {CGO}$ and WGN is shown in Figure 16.

Now, in order to make a fair comparison between the proposed ${ \boldsymbol {P}}_{ \boldsymbol {eso}}$ signal denoising technique and the one which is proposed in [10], the performance of [10] is evaluated on the $Syn \boldsymbol {P}_{ \boldsymbol {eso}}$ signals, which are generated using Equation 11. Then the performances of these two techniques are compared in terms of PRD and runtime at different values of $F$. The runtime of the technique [10] is evaluated in the same computational environment as mentioned before. The result is shown in Table 4.

**TABLE 4:** Comparison of Performance Between the Proposed Technique and [10]

$F$	Mean $PRD$ (%)		Mean runtime in seconds^†^	
	Proposed	[10]	Proposed	[10]
0.1	5.25	5.40	1.49	12.02
0.2	5.36	8.65	1.65	16.45
0.3	8.38	10.85	1.69	20.06
0.4	12.25	12.92	1.85	23.22
0.5	15.10	17.70	1.85	27.69
0.6	18.00	21.63	1.88	31.33
0.7	20.86	24.72	2.03	35.82
0.8	23.82	28.90	2.04	38.76
0.9	26.77	31.56	2.06	42.02
1.0	28.56	34.32	2.10	46.23

^†^ RUNTIMES ARE CALCULATED ON DATA LENGTH OF 1 MINUTE DURATION

From Table 4 it can be seen that the runtime of technique, which is proposed in [10] is much higher compared to the proposed one, and also the PRD values of the proposed $\boldsymbol {P}_{ \boldsymbol {eso}}$ signal denoising technique is smaller compared to [10] for all the values of $F$. Here it is worth mentioning that the run time of [10], which is mentioned in Table 4 comprises only the time required to decompose the ${ \boldsymbol {P}}_{ \boldsymbol {eso}}$ signal using EEMD technique. After decomposition, IMFs are selected based on visual inspection, i.e., manually, and then, those selected IMFs are summed-up to obtain a $\boldsymbol {CGO}$-free ${ \boldsymbol {P}}_{ \boldsymbol {eso}}$ signal.

### A. Clinical Validation

**FIGURE 17.**
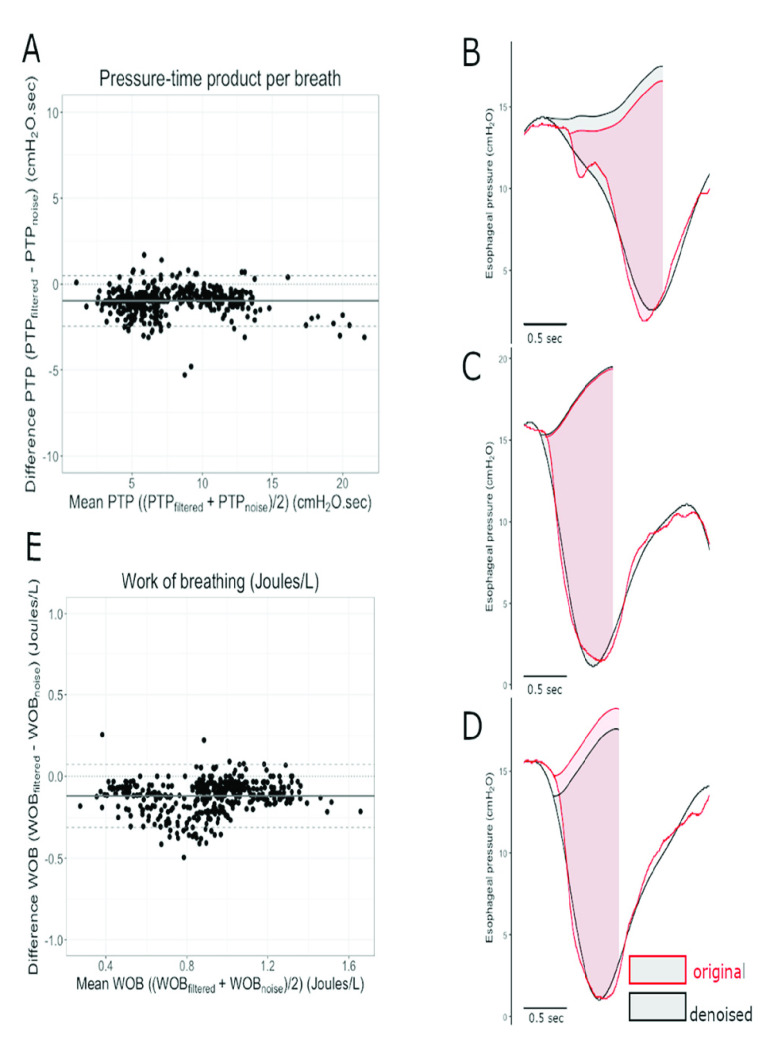
Agreement between parameters of inspiratory effort measured using the noisy and denoised ${P}_{eso}$ signal. A and E: Bland-Altman plots showing agreement between the pressure-time product per breath and work of breathing per liter measured from the denoised signal (PTP/br_filtered_ and WOB$_{\text {filtered}}$) compared to that calculated based on the noisy signal (PTP/br_noise_ and WOB$_{\text {noise}}$). The difference between and the PTP/br_filtered_ or WOB_filtered_ and corresponding PTP/br_noise_ or WOB_noise_ is plotted against the average of the two variables. Black horizontal continuous lines represent mean biases, and dashed lines represent the upper and lower limits of agreement. On the right side of the figure, representative examples of breaths where the PTP/br_filtered_ is higher (B), equal (C) and lower (D) than the PTP/br_noise_ are shown.

A number of parameters that define the mechanics of the patient’s respiratory system such as the elastance of the lung and chest wall, transpulmonary driving pressure, and those, which are needed to quantify the strength of the respiratory effort such as pressure-time product of the respiratory muscles (PTP) and work of breathing (WOB) are calculated based on the esophageal pressure signal [22], [23]). Values of these parameters can significantly be altered by the presence of the cardiac oscillations which modify the amplitude and slope of the esophageal pressure signal. The result can be either an overestimation or underestimation of the real magnitudes of the measured parameters, which can lead to an erroneous clinical judgment. In order to evaluate the performance of the proposed technique on the measurement of clinically relevant parameters, five tracings were selected randomly from patients during unassisted breathing on T-piece, including 425 breaths. These tracings were then denoised using the proposed technique and three parameters namely, PTP per breath, PTP per minute, and WOB were calculated from both the noisy and denoised tracings. PTP per breath was calculated as the integral of the ${ \boldsymbol {P}}_{ \boldsymbol {eso}}$ signal from the beginning of inspiratory effort until the end of inspiratory flow limited by the chest wall recoil pressure (product of tidal volume and chest wall elastance), PTP per breath was calculated as the average PTP per breath for one tracing times the respiratory rate. WOB was calculated as the area enclosed in the ${ \boldsymbol {P}}_{ \boldsymbol {eso}}$-volume loop divided by the tidal volume. The mean (standard deviation) differences between these parameters derived from the original and the denoised tracings are −1.0 (0.8) cm H2O.sec for the PTP per breath, −18.3 (10.6) cm H2O.sec/min for the PTP per minute and −0.1 (0.1) J/L for the WOB representing a relative difference of −15% (11%), −15% (8%), and −11% (11%), respectively. Figure 17 exemplifies the operation.

## V. Conclusion and Discussion

A high efficient, robust and data-driven cardiogenic oscillation removal technique from the esophageal-pressure signal is proposed in this research work. Though the performance evaluation metrics of the proposed techniques are rigorously analyzed in Section III, Figure 16 perhaps better convey the robustness of the technique in removing not only the cardiogenic oscillation, but also the white Gaussian types of noises. A very important factor about the proposed technique is that its performance does not depend on any predefined mathematical-model or function unlike the wavelet transform. Another advantage of the proposed technique is that the singular spectrum analysis parameters are made adaptive to the sampling rate of the signal, and therefore it is not required to change the parameters manually if the sampling rate of the signal alters. The proposed technique can also be easily adapted for denoising other biomedical signals such as the electrocardiogram and photoplethysmogram, which exhibit periodic or quasi-periodic nature.

The performance of the proposed technique is tested on 75 esophageal-pressure signals, which are collected from the intensive care unit of the St. Michael’s Hospital, Toronto, Ontario, Canada, and also on 1800 signals which are generated by adding the synthetic esophageal-pressure signals with real cardiogenic-oscillation noises. Both the quantitative and qualitative distortion measure metrics show that the proposed denoising technique is robust enough to expel out the cardiogenic-oscillation noises efficiently. The main reasons for achieving such an attractive denoising performance are: (i) choosing the optimum value of the window-length, (ii) enhanced covariance among the eigenvector, (iii) implementing the bandpass and notch filtering operations at the reconstruction-component levels, and (iv) the singular spectrum analysis technique captures the periodicity of the oscillatory modes of the esophageal-pressure signal better than that of the fixed filtering-based approaches. Figure 15 shows a comparison of the denoising performance between the proposed technique and fixed-filtering approaches. The resulting signals suggest that the proposed technique outperforms fixed-filtering techniques in removing $\boldsymbol {CGO}$ interference. Figures 6 to 8 show that the proposed technique is efficient enough to process the esophageal-pressure signals of different morphologies.

At its present setting, the proposed technique is applied on a full-length esophageal-pressure signal at once. However, the technique can also be adapted to use in real-time applications. Formation of a $M\times N$ Henkel matrix is the foremost criteria of a singular spectrum analysis-based method. In order to process an esophageal pressure signal using the proposed technique, following Equation 2, the number of columns of the Henkel matrix should be $N=1.5\times sampling\,\,rate\,\,(Hz)$. Hence, at least $N+1$ numbers of samples are required in order to form the Henkel matrix, and in real-time application the proposed technique can be applied iteratively on $N+1$ number of samples. For an example, if the sampling rate of the esophageal pressure signal is 200 Hz, then the minimum number of samples, which is required to process using the proposed technique, is $(1.5\times 200)+1=301$. It is observed that the average processing time of 301 samples is ~0.025 seconds. A real-time implementation of this proposed denoising technique can help providing a precise estimation of the respiratory mechanics and breathing effort. Moreover, it can also be applied in existing monitoring devices, such as ventilators, for the clinicians to calculate these parameters correctly in real-time allowing a personalized management of ventilator settings and sedation to avoid harm.

From a clinical perspective, we showed that the denoised signal allows for calculation of clinically important parameters derived from ${ \boldsymbol {P}}_{ \boldsymbol {eso}}$. PTP and WOB correlate with energy expenditure of the respiratory muscles [24] and allow for estimation of the risk of patient self-inflicted lung injury and myotrauma [25], [26]. The relative and absolute differences between the parameters calculated with the noisy and denoised signal are small and clinically acceptable; the precision (i.e. distribution of the difference) is also acceptable. The results show that, in average, the parameters calculated by the denoised signal are slightly lower than that of the noisy signal. However, upper limit of agreement (mean +1.96 SD) crosses zero suggesting that for some breaths the use of our denoising technique results estimation of higher measures of inspiratory effort and for others in a lower effort compared to the noisy signal. To tease out if there is an overall slight overestimation of the real measurement of inspiratory effort by the noisy signal or slight underestimation by the denoised signal, an independent comparison with other measures of muscular activity such as energy consumption should be performed in future studies. Only one level of pressure support; 5 cm H_2_O, was tested in the research which is considered as a relatively low level of support in the clinical setting.

The result of the subjective assessment i.e., the qualitative measure provides a better insight into the quality of the denoised esophageal-pressure signals as the assessment is conducted by the field-experts. As per the mean opinion score error criteria the denoised esophageal-pressure signals (overall) and also all of its features fall under the category ‘very good’.
